# Percutaneous Curettage and Continuous Irrigation for MRSA Lumbar Spondylodiscitis: A Report of Three Cases

**DOI:** 10.1155/2009/253868

**Published:** 2009-05-26

**Authors:** Yoshiki Yamagami, Sei Shibuya, Satoshi Komatsubara, Tetsuji Yamamoto, Nobuo Arima

**Affiliations:** Department of Orthopaedic Surgery, School of Medicine, Kagawa University, 1750-1 Ikenobe, Miki-cho, Kagawa 761-0793, Japan

## Abstract

There has been a recent increase in pyogenic spondylitis caused by methicillin-resistant *Staphylococcus aureus* (MRSA) associated with an increasing number of compromised patients. As long as serious paralysis is absent, we recommend percutaneous curettage and continuous irrigation as an effective treatment for MRSA lumbar spondylodiscitis. Under local anesthesia, the affected lumbar discs were curetted using percutaneous nucleotomy, and tubes were placed for continuous irrigation. The period of continuous irrigation was generally 2 weeks. Infection was controlled after one procedure in two cases and after two procedures in one case. Postoperative radiography and magnetic resonance imaging (MRI) showed callus formation, normalized signal intensity in vertebral bodies, and regression of abscesses. Open surgery under general anesthesia has been considered risky in patients with poor performance status or old age. The present method, which is an application of needle biopsy, can be performed under local anesthesia and is minimally invasive.

## 1. Introduction

Recently, pyogenic spondylitis has become more common as an opportunistic infection, accompanying an increase in compromised hosts due to aging and underlying diseases such as diabetes mellitus and malignant tumors [1]. Pyogenic spondylitis commonly occurs in the lumbar vertebrae of males from the middle age; hematogenous spread of pathogenic bacteria such as *Staphylococcus aureus* results in infection of the intervertebral disc tissue and adjacent vertebral bodies, causing acute onset of severe local pain and high fever [2]. However, depending on the type of pathogenic bacteria, pain may be mild and fever may be low-grade or absent, resulting in delayed diagnosis and chronic progression of the disease [3]. While the advent of magnetic resonance imaging (MRI) has facilitated diagnosis, there are still cases that cannot be detected by diagnostic imaging soon after onset and are diagnosed only when neurological symptoms appear. 

In particular, methicillin-resistant *Staphylococcus aureus* (MRSA) and multidrug resistant bacteria are often refractory to treatment. We encountered 3 cases of pyogenic lumbar spondylodiscitis caused by MRSA, which were successfully managed by percutaneous curettage using percutaneous nucleotomy techniques [4] supplemented with continuous irrigation.

## 2. Surgical Procedures and Medical Manegement

Percutaneous curettage was performed under local anesthesia with the patient in a lateral decubitus position, following the technique of percutaneous nucleotomy. Hijikata's percutaneous nucleotomy device (Tanaka Medical Instruments, Tokyo, Japan) was used. A probe was inserted at a point 8-9 cm lateral to the midline, passing through the paraspinal muscles to reach the intervertebral disc. Curettage of the lesion site was done using the herniation forceps. Then the lesion was irrigated with a large volume of physiological saline (2000–3000 mL). For continuous irrigation, an epidural anesthetic tube for infusion of irrigation fluid and a 3 mm diameter plastic tube for drainage were placed in the intervertebral disc space. Continuous irrigation was performed using physiological saline containing heparin (400 mL/23 h) and physiological saline containing vancomycin (VCM; 0.5 g/100 mL/1 h) for 14 days. Two to three days before continuous irrigation was discontinued, bacteriological culture of the irrigation fluid was conducted. In all three cases, continuous irrigation was terminated after confirming that the irrigation fluid was culture negative.

Since all three cases had positive blood or tissue culture yielding MRSA, systemic VCM (1 g/day) was given for 4 to 6 weeks after curettage, with regular monitoring of blood levels. The minimum inhibitory concentration of vancomycin for MRSA was 2 *μ*g/mL in Case 1, and 1 *μ*g/mL in Cases 2 and 3. In Case 1, after systemic VCM was terminated, oral levofloxacin (LVFX) (300 mg/day) was continued for 3 months. In Cases 2 and 3, after systemic VCM was terminated, oral trimethoprim/sulfamethoxazole (TMP/SMX; 800 mg/day) and rifampicin (RFP; 450 mg/day) were continued for approximately three months.

## 3. Case Presentation

### 3.1. Case 1: A 49-Year-Old Man

 The patient had a three-year history of malignant rheumatoid arthritis accompanied by steroid-induced diabetes. His respiratory function was lowered due to interstitial pneumonia. Three months before diagnosis, he visited our department because of fever at 39°C together with redness and swelling of the right olecranal region and the right crus. Imaging studies detected an abscess at the corresponding sites. Furthermore, blood culture yielded MRSA. After the wound was healed, fever recurred and abdominal pain appeared. Abdominal computed tomography (CT) led to a diagnosis of left iliopsoas abscesses. A radiologist inserted a drainage tube under CT guidance to drain the abscess. After several days, the patient reported strong lumbar pain and pyogenic spondylodiscitis was diagnosed based on radiographic and MRI findings.

**Figure 1:**
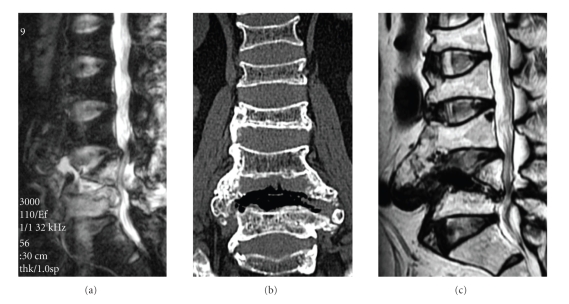
Imaging studies for Case 1. (a) Preoperative lumbar spine MRI showing sagittal T2-weighted image. Destruction of the L4 vertebral body and an abscess are evident. (b) Postoperative lumbar spine MRI showing sagittal T2-weighted image. Normalization of signal intensity in vertebral bodies and disappearance of abscess are observed. (c) Postoperative CT showing callus formation bridging the vertebral bodies around the L4/5 intervertebral region.

Preoperative clinical evaluation showed only lumbar pain without obvious neurological defects. Radiography revealed marked destructive changes of the L4 vertebral body. On MRI, an abscess with high signal intensity in the L4/5 intervertebral region was seen on T2-weighted images (Figure 1(a)), while the L4 and L5 vertebral bodies as a whole showed low signal intensity on T1-weighted images. We conducted percutaneous debridement followed by continuous irrigation for 14 days. A preoperative blood test showed C-reactive protein (CRP) 4.66 mg/dl, white blood cell count (WBC) 6700/mm^3^, alkaline phosphatase (ALP) 262 IU/dl, and lactate dehydrogenase (LDH) 515 IU/dl. CRP decreased to 0.89 mg/dl on the 14th postoperative day, and was normalized at the 3rd week after the procedure. Thereafter, although CRP sometimes increased to 4–6 mg/dl due to the effect of malignant rheumatoid arthritis, marked CRP elevation and worsening of lumbar pain were not observed. 

At 3 months after curettage, a plain radiogram indicated ossification with osteosclerosis, and MRI showed normalization of signal intensities in the L4 and L5 vertebral bodies as well as marked reduction in abscess size. At 12 months, plain radiography and CT showed bridging callus between the vertebral bodies (Figure 1(b)). MRI revealed normalization of signal intensity in the vertebral bodies and resolution of abscesses (Figure 1(c)). MRSA infection was thus considered cured.

### 3.2. Case 2: A 53-Year-Old Man

The patient had a history of schizophrenia, diabetes, and hyperthyroidism. Due to the mental disorder, no attempt was made to control blood glucose, and angina attack was also documented. Fever and severe lumbar pain developed 4 months before presentation, and pyogenic spondylitis was diagnosed in another hospital. Debridement of the infected focus using percutaneous nucleotomy techniques under local anesthesia and placement of a drainage tube were performed. However, the patient developed fever and markedly intensified lumbar pain 3 months after surgery. He therefore presented at our institution and was admitted.

 On admission, lumbar pain was so severe that the patient had difficulty maintaining a sitting position for 5–10 minutes. Radiating pain and reduced muscle strength [level 3-4 on manual muscle test (MMT)] suggested L5 nerve root lesion. Plain radiography revealed marked bone destruction and osteolytic changes of the L5 vertebral body, and the vertebral body height was reduced by approximately three-quarters. A cavity was found within the L4 vertebral body. MRI revealed a hyperintense abscess at the L4/5 intervertebral region on T2-weighted imaging, while both L4 and L5 were clearly hypointense on T1-weighted imaging. We performed percutaneous debridement followed by continuous irrigation for 14 days. A preoperative blood test showed CRP 5.82 mg/dl, WBC 7800/mm^3^, ALP 451 IU/dl, and creatine phosphokinase (CPK) 188 IU/dl. CRP decreased to 0.75 mg/dl on the 14th postoperative day, and was normalized at 4 weeks after curettage. Thereafter, no elevation was observed.

 Bacteriological culture of tissues removed during curettage confirmed MRSA infection. At 3 months after the procedure, plain radiography showed clear ossification, and MRI showed recovery of signal intensity in the L4 and L5 vertebral bodies and marked reduction of abscesses, indicating that infection was controlled. Pyogenic spondylodiscitis was considered cured.

 The pain in the left lower extremities observed before curettage subsequently remitted, but became exacerbated at 7 months postoperatively. Although lumbar spinal canal stenosis accompanied by vertebral deformation was suspected, neurological symptoms disappeared after performing partial laminectomy at L4/5. At the last follow-up 29 months after surgery, plain radiography and CT showed ossification and continuity of the vertebral bodies due to osteogenesis. MRI revealed normalization of signal intensity of the vertebral bodies and resolution of abscess. Blood testing showed no abnormalities. MRSA infection was thus considered cured.

### 3.3. Case 3: A 70-Year-Old Man

Diabetes was diagnosed 7 years earlier, and the patient had been receiving subcutaneous injections of insulin. The patient was admitted to the Department of Ophthalmology for elective cataract surgery. On day 2 after admission, heart failure symptoms worsened and the patient was transferred to the Department of Cardiovascular Medicine. Acute exacerbation of heart failure necessitated endotracheal intubation, and total management with a ventilator was continued for approximately 5 days. Infectious endocarditis was not detected. After extubation, both sputum and blood cultures yielded MRSA. The patient developed severe lumbar pain. He was referred to our department 7 weeks after admission. 

Preoperative clinical evaluation showed only lumbar pain and no obvious neurological symptoms. Plain radiography revealed intervertebral disc narrowing and end plate destruction at L1 and L2 vertebral bodies. MRI revealed a hyperintense abscess extending from the L1/2 intervertebral region to the anterior side of the vertebral bodies on T2-weighted imaging, and a hypointense region involving both vertebral bodies on T1-weighted imaging. In addition, many abscesses were found within bilateral iliopsoas muscles. On day 2 after diagnosis, percutaneous curettage was performed using percutaneous nucleotomy techniques supplemented with continuous irrigation. As preoperative evaluation showed severe angina pectoris concomitant with impaired coronary circulation, curettage was performed without discontinuing anticoagulant therapy. In contrast to Cases 1 and 2, a 3-mm diameter plastic tube for infusion of irrigation fluid and a 5-mm diameter plastic tube for drainage were placed in the intervertebral region for continuous irrigation. The original schedule was to continue irrigation for 2 weeks, but the patient exhibited hyperactivity and abnormal behavior accompanying cerebrovascular dementia, and he pulled out the irrigation tubes by himself on day 2, thereby terminating the irrigation. Treatment was considered inadequate and a second procedure using the same methods was conducted 2 weeks after the initial curettage. Continuous irrigation was maintained for 14 days after the second procedure. A preoperative blood test showed CRP 6.87 mg/dl, and WBC 14300/mm^3^. CRP decreased to 0.75 mg/dl at 4 weeks after the first procedure, and was normalized at 7 weeks, with no elevation thereafter.

Imaging evaluation at 12 months after the second procedure showed decreased hypointensity on T1-weighted imaging and reduced hyperintensity together with the disappearance of bilateral iliopsoas abscesses on T2-weighted imaging. On CT, although slight collapse of the vertebral body had progressed, fusion between vertebral bodies had been achieved, callus formation and ossification were evident. MRSA infection was thus considered cured.

## 4. Discussion

With regard to the application of needle biopsy in pyogenic spondylodiscitis, a drainage tube has been placed in various reports [5]. Particularly, CT-guided percutaneous drainage is a minimally invasive procedure and can be performed safely. However, drainage tubes needed to be exchanged in 21 of 33 patients in one study [5]. Furthermore, when a tube is placed for long periods of time, there is a risk of retrograde infection. 

 Surgical treatment and instrumentation surgery have been used actively for the management of spinal osteomyelitis [1]. Insertion of a titanium mesh cage between vertebral bodies and pedicle screw fixation has been reported to achieve good results [6, 7]. However, although spinal reconstruction can correct deformities and lead to robust fixation, the long operative time and greater blood loss make this procedure highly invasive, irrespective of the use of a one- or two-stage procedure. There has even been a report of surgical death [8]. Instrumentation surgery is thus a high-risk procedure for compromised patients. 

Our method based on percutaneous nucleotomy techniques for pyogenic spondylitis was first reported by Yu et al. [9], and Gill and Blumenthal [10]. This method has the advantages of being a minimally invasive procedure that is performed under local anesthesia, and requires a small incision of less than 10 mm. The sample obtained during curettage can be used in bacteriological and histopathological investigations. Furthermore, supplementation with continuous irrigation allows simultaneous administration of local antibiotics and drainage [11, 12]. Another advantage is that in cases where cure is not achieved or infection recurs, the same treatment can be repeated. Previous study has indicated that even in the presence of epidural and paravertebral abscesses, curettage of the lesion in lumbar discs followed by continuous irrigation results in reduction and disappearance of surroundings lesions [12]. In our three cases also, surrounding abscesses were eventually resolved. If diagnosis can be made soon after disease onset, early treatment by this method may minimize destruction of the vertebral body or intervertebral disc, which may also shorten the treatment period. As a similar technique, several studies have reported transpedicular intervertebral disc curettage [13, 14], but this procedure requires treatment of the pedicle so that it may be difficult to perform under local anesthesia. In addition, medial displacement from the pedicle may damage the neural tissue.

In general, percutaneous curettage is considered to be indicated for patients with lesions localized to one intervertebral region, little vertebral body destruction resulting in mild spinal alignment abnormality, and no serious paralytic symptoms [12]. However, treatment of our 3 cases using percutaneous curettage and continuous irrigation achieved rapid improvement of general status, including pain and fever, while imaging studies confirmed bone formation and union. In addition, the irrigation solution did not leak, circulatory failure did not occur, and no side effects were seen during the procedure. The efficacy demonstrated for MRSA pyogenic spondylodiscitis, which is drug-resistant and refractory to treatment and results in marked vertebral body destruction, suggests that this method may be suitable as an effective treatment. Although medical therapy alone could also be curative, direct approach to the lesion by the present method may resolve clinical manifestations more rapidly in cases with marked bone destruction and abscess formation. Furthermore, even with advanced disease, as long as serious paralysis (MMT level 2 or below) is absent, attempting this method as the first surgical treatment is preferable to open surgery.
